# Association between lifestyle and combined general and abdominal obesity in Korean adults during the pre-pandemic and pandemic periods: using the Korea National Health and Nutrition Examination Survey (2019–2020)

**DOI:** 10.1186/s12889-026-26884-6

**Published:** 2026-03-28

**Authors:** Doyeon Kim, Yeon Hee Woo

**Affiliations:** 1 Division of Health and Nutrition Survey and Analysis, Department of Chronic Disease Prevention and Control, Korea Disease Control and Prevention Agency, Cheongju, Republic of Korea; 2 Department of Nursing, Chodang University, Muan, Republic of Korea

**Keywords:** Obesity, Combined general and abdominal obesity, COVID-19, South Korea

## Abstract

**Background:**

The COVID-19 pandemic has profoundly influenced lifestyle behaviors and increased prevalence of obesity worldwide, with varying effects across socioeconomic groups. We aimed to examine the association between lifestyle and combined general and abdominal obesity between 2019 (pre-pandemic period) and 2020 (pandemic period) by income level in South Korea.

**Methods:**

We used data from the 8th Korea National Health and Nutrition Examination Survey (KNHANES) in 2019 and 2020. We compared differences in demographic and lifestyle variables during pre-pandemic and pandemic period, considering income level (high and low) and obesity type (normal weight, general obesity, abdominal obesity, and combined general and abdominal obesity). Logistic regression analysis was conducted to examine the association between lifestyle and combined general and abdominal obesity stratified by income level.

**Results:**

The prevalence of combined general and abdominal obesity was higher during pandemic period than during pre-pandemic period. During pandemic period, the alcohol consumption in the normal-weight and high perceived stress in the abdominal obesity were lower, while strength training in the combined general and abdominal obesity was higher than during pre-pandemic period in the low-income group. In contrast, in the high-income and normal-weight groups, walking was lowered, and the skipping breakfast was higher during pandemic period than during pre-pandemic period. In the high-income group, lack of strength training, perceived stress, and excessive energy intake were associated with combined general and abdominal obesity.

**Conclusions:**

These results suggest that modifiable behavioral variables such as physical activity, dietary factors, and stress were associated with combined general and abdominal obesity in high-income level during the pandemic period. Our findings underscore the importance of these variables as core strategies for obesity prevention.

## Background

Obesity prevalence has increased substantially worldwide since 1990, with one in eight people classified as obese in 2022 [1]. In response, the World Health Organization (WHO) has recognized obesity as a disease and proposed global actions for chronic disease prevention and management [2, 3]. South Korea has also prioritized obesity control in the National Health Promotion Comprehensive Plan 2030 [4]. Nevertheless, obesity remains a growing public health concern [5]. In Korea, national estimates indicate that body mass index (BMI)-defined general obesity increased from 29.7% in 2009 to 37.2% in 2023 [6, 7], while waist circumference (WC)-defined abdominal obesity increased from 22.9% to 33.3% over the same period [6]. These trends underscore the need to consider both general and abdominal obesity when assessing obesity-related health risks.

General obesity and abdominal obesity both reflect excess adiposity and cardiometabolic risk, but they capture related yet distinct aspects of these processes [8]. As both phenotypes have increased, greater attention has been drawn to individuals who meet criteria for both. In this study, we refer to combined general and abdominal obesity as the coexistence of BMI-defined general obesity and WC-defined abdominal obesity. This phenotype has been associated with higher risks of cancer [9], type 2 diabetes [10], and cardiovascular disease [11] than other obesity patterns. However, epidemiological evidence on lifestyle correlates of combined general and abdominal obesity remains limited, and determinants may differ across obesity phenotypes [12].

Lifestyle behaviors are fundamental determinants of health [13]. They include behavioral variables such as physical activity, diet, smoking, and alcohol consumption that are closely associated with obesity [14]. These behaviors are not merely individual choices but are fundamentally shaped by broader socioeconomic conditions [15]. In South Korea, socioeconomic indicators such as income and educational level have been reported to be strongly associated with obesity [16]. Among these, income level is related to nutritional status, food intake, and disease morbidity [16, 17]. Individuals with higher socioeconomic status tend to have better nutritional conditions [16], whereas low-income groups often face a disproportionate obesity burden, a trend historically linked to unhealthy dietary patterns and limited opportunities for physical activity [18]. Therefore, examining lifestyle variables by obesity phenotype while considering income level may help clarify income-related inequalities relevant to obesity prevention.

The COVID-19 pandemic may have further accelerated obesogenic environments. Longitudinal evidence has reported modest average increases in weight and BMI during 2020–2021 [19]. Social changes due to quarantine affected lifestyle and dietary behaviors [20]. Reduced opportunities for physical activity and increased sedentary time have been widely reported [21]. These changes may have contributed to weight gain and an increased risk of obesity-related comorbidities [22, 23]. In addition, the economic impact of the COVID-19 pandemic has remained substantial among low-income households [24], potentially reinforcing disparities in obesity-related behaviors.

In this study, we used data from the 8th Korean National Health and Nutrition Examination Survey (KNHANES), conducted in 2019 (pre-pandemic period) and 2020 (pandemic period). We compared lifestyle behaviors across obesity phenotypes between the 2019 and 2020 survey years, stratified by income level. We also examined the associations between lifestyle variables and combined general and abdominal obesity among adults in South Korea.

## Methods

### Ethics statement

The KNHANES 2019 and 2020 were conducted under the approval of the Research Ethics Review Committee of the Korea Disease Control and Prevention Agency (approval numbers: 2018-01-03-C-A and 2018-01-03–2 C-A, respectively). To conduct this study, raw data and data analysis guidelines for 2019 and 2020 were acquired from the official KNHANES database (Korea Disease Control and Prevention Agency) for the purpose of this study.

### Data

This cross-sectional study used raw data from the KNHANES conducted from 2019 to 2020. To ensure clarity in our comparative analysis, we defined the 2019 KNHANES dataset as the pre-pandemic period and the 2020 dataset as the pandemic period. It is important to note that these datasets consist of independent cross-sectional samples; therefore, the results represent population-level trends rather than individual longitudinal shifts. Additionally, while the intensity of COVID-19 restrictions varied across 2020, this study evaluates the cumulative annual impact. We acknowledge that our analysis cannot distinguish the effects of specific, time-bound policy measures or the exact timing of lockdown phases on lifestyle behaviors.

**Fig. 1:**
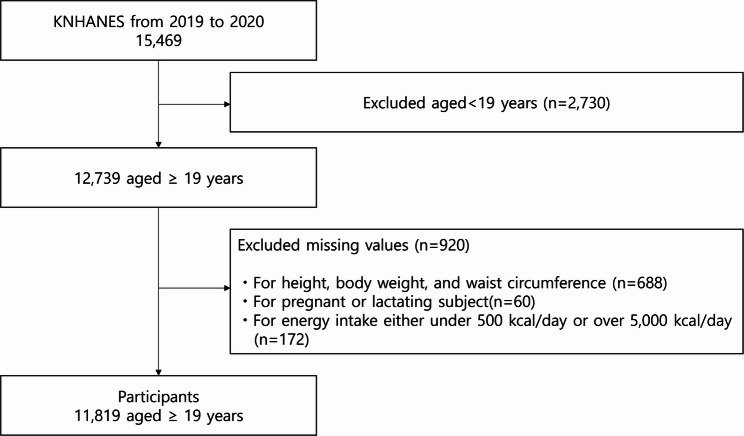
Sampling process. KNHANES, Korea National Health and Nutritional Examination Survey

In this study, the participants were adults aged ≥ 19 years who responded to a health questionnaire and physical examination survey from 2019 to 2020. Of the initial 15,469 participants, a final sample of 11,819 was included in the analysis after excluding: those with missing values of questions and physical examinations (height, weight, and WC), pregnant or breastfeeding women, and those with energy intakes below 500 kcal/day or > 5,000 kcal/day [25] (Fig. 1).

### Classification of obesity

BMI and WC were measured to determine the combined general and abdominal obesity. To measure general obesity, BMI was calculated using the following formula: BMI = body weight (kg)/height (m)^2^. BMI was classified into the following categories: underweight (< 18.5 kg/m^2^), normal weight (≥ 18.5 kg/m^2^ and < 23 kg/m^2^), overweight (≥ 23 kg/m^2^ and < 25 kg/m^2^), or obese (≥ 25 kg/m^2^) [26]. The participants were classified into non-obese (underweight, normal-weight, and overweight) and obese groups. Abdominal obesity was assessed using WC. A WC ≥ 85 cm in women and ≥ 90 cm in men indicates abdominal obesity [27]. Obesity types were categorized into four groups: normal (neither general nor abdominal obesity), general obesity without abdominal obesity, abdominal obesity without general obesity, and combined general and abdominal obesity [10] (Table 1).

**Table 1:** Definitions of obesity phenotypes based on BMI and WC

Category	BMI criterion	WC Criterion
Normal (Neither general nor abdominal obesity)	BMI < 25 kg/㎟	WC < 90 cm(men) / < 85 cm(women)
General obesity (only) (without abdominal obesity)	BMI ≥ 25 kg/㎟	WC < 90 cm(men) / < 85 cm(women)
Abdominal obesity (only) (without general obesity)	BMI < 25 kg/㎟	WC ≥ 90 cm(men) / ≥85 cm(women)
Combined general and abdominal obesity	BMI ≥ 25 kg/㎟	WC ≥ 90 cm(men) / ≥85 cm(women)

BMI cut-off(≥ 25 kg/㎟) and WC cut-offs(men≥90 cm, women≥85 cm) were defined according to the clinical practice guidelines of the Korean Society for the Study of Obesity (KSSO) [26, 27]

*BMI* body mass index, *WC* waist circumference

### General characteristics

Sex, age, height, weight, BMI, WC, educational level, marital status, income level, and region data from the KNHANES were analyzed. Age, height, weight, BMI, and WC were analyzed as means ± standard errors (SEs). Educational level was categorized as elementary school, middle school, high school, or college. Marital status was categorized as married or single. Socioeconomic household status was divided into four quartiles (lowest, lowest-middle, upper-middle, and highest). For the stratified analysis, income levels were further grouped into two categories: low-income group (lowest and lowest-middle quartiles) and high-income group (upper-middle and highest quartiles). Region was divided into urban and rural. For logistic regression analysis, age was categorized into three groups: young adults (≤39 years), middle-aged adults (40–64 years), and older adults (≥65 years). Chronic diseases were categorized as “yes” if there was hypertension, diabetes, or hypercholesterolemia, and “no” if these were not present.

### Health behavior characteristics

To investigate the health behaviors of the study participants, their smoking and alcohol consumption behaviors were analyzed. Smoking was classified based on the ‘current smoking rate’, with ‘past smoking’ and ‘non-smoking’ categorized as “no” and current smoking as “yes”. Alcohol consumption was classified based on the ‘monthly drinking rate’, with ‘lifetime non-drinkers and those who consumed less than one alcoholic drink per month in the past year’ categorized as “no” and ‘those who consumed at least one alcoholic drink per month’ in the past year categorized as “yes”.

### Physical activity characteristics

Walking, strength training, aerobic exercise, and sedentary behavior were analyzed to investigate the physical activity characteristics of the participants. Walking was assessed using the item ‘days of walking per week’ categorized as: “never,” “1–2 days/week”, “3–4 days/week” and “5–7 days/week”. Strength training was assessed using ‘days of strength training per week’ classified based on weekly participation, with no strength training categorized as “no” and at least one day of strength training categorized as “yes”. Aerobic exercise was assessed using the Global Physical Activity Questionnaire, which captures activity in the occupational, transportation, and leisure-time domains. For the purpose of this study, activity levels from all three domains were integrated to evaluate the total weekly duration of moderate-to-vigorous physical activity. Those who engaged in at least 2 h and 30 min of moderate-intensity physical activity, at least 1 h and 15 min of vigorous-intensity physical activity, or an equivalent combination of both were categorized as “yes”, whereas those who did not meet these criteria were categorized as “no”. Sedentary behavior was assessed using ‘average daily sitting time(hours/day)’ categorized as “≤ 8 hours” and “> 8 hours”.

### Dietary characteristics

Diet was categorized as eating out and skipping breakfast, and energy, fat, and carbohydrate intake percentages. Eating out was assessed using ‘average eating-out frequency during the past year’ and categorized as “3 times or less/month”, “1–6 times/week”, and “every day.” Breakfast skipping was derived by reverse-coding ‘frequency of breakfast consumption per week during the past year’ and categorized as “≤2 days/week,” “3–6 days/week,” and “every day.” Energy, fat, and carbohydrate intakes were operationalized using KNHANES nutrient variables and expressed as ratios (and corresponding percentages). For logistic regression analyses, each variable was categorized into three levels: insufficient, adequate, and excessive. Total energy intake was obtained as ‘kcal/day’ and was evaluated relative to the Estimated Energy Requirement (EER) based on the Dietary Reference Intakes for Koreans (KDRIs, 2015) using age- and sex-specific standards. Energy adequacy was calculated as: (energy intake*/ EER) × 100*. The energy intake-to-EER ratio (energy intake /EER) was classified as insufficient (< 0.75), adequate (0.75–1.25), and excessive (> 1.25) (i.e., > 125% of EER) [28]. Fat intake was obtained as fat intake (g/day) and expressed as the fat energy ratio (% energy) using standard conversion factors: (fat intake × 9) / ((protein intake × 4) + (fat intake × 9) + (carbohydrate intake × 4)) × 100. The ratio of fat intake was classified as “insufficient” (< 0.15), “adequate” (0.15–0.30), and “excessive” (> 0.30). Carbohydrate intake was obtained as carbohydrate intake (g/day) and expressed as the carbohydrate energy ratio (% energy) as: (carbohydrate intake × 4) / ([protein intake × 4] + [fat intake × 9] + [carbohydrate intake × 4]) × 100. The ratio of carbohydrate intake was categorized as “insufficient” (< 0.55), “adequate” (0.55–0.65), and “excessive” (> 0.65) [29].

### Psychological characteristics

Psychological characteristics were evaluated by assessing perceived stress using the variable ‘perceived stress level.’ Perceived stress was dichotomized as “little” versus “a lot.”

### Statistical analysis

All analyses were conducted using IBM SPSS Statistics (version 29.0; IBM Corp., Armonk, NY, USA) to account for the complex, stratified, multistage probability sampling design. The analysis incorporated the stratification variable, primary sampling unit, and sample weight to ensure nationally representative estimates.

Continuous variables are presented as weighted means and SEs using the complex sample general linear model, while categorical variables are summarized as frequencies (n) and proportions (%) using cross-tabulation analysis. To compare characteristics during the pre-pandemic and pandemic periods, categorical variables were analyzed with the Rao–Scott adjusted chi-squared test, and continuous variables were compared using the complex sample general linear model.

To examine the association between lifestyle variables and combined general and abdominal obesity during the pre-pandemic and pandemic period, complex sample logistic regression analyses were performed after adjusting for sex and age, as both variables are well-established demographic determinants of obesity and lifestyle behaviors [1, 30], and the results are expressed as odds ratios (ORs) and 95% confidence intervals (CIs). Separate models were fitted for each lifestyle variable to estimate its association with combined general and abdominal obesity (i.e., each model included one lifestyle variable as the exposure and was adjusted for age and sex). To ensure the robustness of our logistic regression models, we performed multicollinearity diagnostics using Variance Inflation Factors (VIF); all variables showed VIF values < 10, confirming the absence of significant collinearity between dietary and lifestyle variables. Regarding the risk of type I error due to multiple comparisons, we focused on reporting exact p-values and 95% CIs rather than applying a universal p-value correction. This approach was chosen to maintain the power of this exploratory study in identifying various behavioral patterns during the pandemic. However, the results were interpreted cautiously, with a focus on clinical relevance and effect sizes (ORs) rather than statistical significance alone.

## Results

The sociodemographic characteristics of adults aged ≥ 19 years from the KNHANES during pre-pandemic period and during pandemic period are presented in Table 2. The number of participants aged ≥ 19 years was 6,098 and 5,721 during pre-pandemic and pandemic period, respectively. Among these, 2,722 were men and 3,376 were women during pre-pandemic period and 2,590 were men and 3,131 were women during pandemic period. The overall distributions of sex, age group, education, marital status, household income, region, and major health behaviors were broadly similar between survey years. However, measures related to adiposity, including body weight, BMI, WC, and the prevalence of combined general and abdominal obesity were higher during pandemic period than during pre-pandemic period. During the pandemic period, walking and carbohydrate intake were lower, whereas fat intake was higher than during the pre-pandemic period (Table 2).

**Table 2:** Socio-demographic characteristics and lifestyle among Korean adults in the pre-pandemic and pandemic periods

Variable	Characteristics	Pre-pandemic	Pandemic	*P*
		*n* (%) or M ± SE	*n* (%) or M ± SE	
Obesity type	Normal	3,521(59.1)	3,100(56.0)	< 0.005
	General obesity	379(7.3)	376(7.2)	
	Abdominal obesity	508(7.4)	454(6.4)	
	Combined general and abdominal obesity	1,690(26.3)	1,791(30.5)	
General characteristics				
Sex	Man	2,722(50.0)	2,590(50.1)	0.869
	Woman	3,376(50.0)	3,131(49.9)	
Age (years)		48.26 ± 0.49	48.33 ± 0.54	0.930
Height (cm)		165.13 ± 0.19	165.17 ± 0.22	0.876
Body weight (kg)		65.43 ± 0.24	66.42 ± 0.29	0.008
BMI (kg/m^2^)		23.88 ± 0.07	24.20 ± 0.08	0.003
WC (cm)		83.62 ± 0.22	84.37 ± 0.21	0.017
Education	≤ Elementary school	1,058(12.9)	870(11.4)	0.469
	Middle school	551(7.6)	515(7.6)	
	High school	1,934(35.5)	1,850(37.8)	
	≥ College	2,211(44.0)	2,008(43.3)	
Marital status	Married	4,175(86.0)	3,705(84.8)	0.340
	Unmarried	879(14.0)	861(15.2)	
Household income	Lowest	1,191(15.0)	1,063(14.9)	0.473
	Lowest middle	1,565(24.9)	1,347(22.1)	
	Upper middle	1,533(27.1)	1,576(28.8)	
	Highest	1,777(33.0)	1,711(34.2)	
Region	Urban	4,880(84.7)	4,548(86.6)	0.608
	Rural	1,218(15.3)	1,173(13.4)	
Chronic disease	No	2,998(56.9)	2,728(56.0)	0.602
	Yes	2,901(43.1)	2,779(44.0)	
Health behavior characteristics				
Smoking	No	4,955(80.3)	4,734(81.8)	0.177
	Yes	1,057(19.7)	935(18.2)	
Alcohol consumption	No	2,768(42.8)	2,775(45.3)	0.083
	Yes	3,250(57.2)	2,896(54.7)	
Physical Activity characteristics				
Walking	Never	1,012(15.7)	933(16.1)	0.014
	1–2days/week	869(14.4)	869(17.4)	
	3–4days/week	1,191(20.0)	1,056(20.6)	
	5–7days/week	2,681(49.9)	2,386(45.9)	
Strength training	No	4,301(72.4)	3,857(71.1)	0.286
	Yes	1,457(27.6)	1,388(28.9)	
Aerobic exercise	No	3,261(54.2)	3,021(55.1)	0.550
	Yes	2,489(45.8)	2,220(44.9)	
Sedentary behavior	≤ 8 h	2,317(38.2)	2,127(38.0)	0.926
	> 8 h	3,387(61.8)	3,082(62.0)	
Dietary characteristics				
Eating out	3 times or less/month	1,416(21.1)	1,430(26.0)	0.002
	1–6 times/week	2,664(51.0)	2,148(51.1)	
	Every day	1,198(27.9)	838(23.0)	
Breakfast skipping	≤ 2 days/week	3,369(57.2)	2,716(54.1)	0.014
	3–6 days/week	1,102(24.5)	872(23.4)	
	Every day	807(18.3)	828(22.4)	
Energy intake %		90.27 ± 0.74	89.42 ± 0.73	0.416
Fat intake %		22.52 ± 0.21	23.63 ± 0.26	0.002
Carbohydrate intake %		61.83 ± 0.27	60.76 ± 0.32	0.016
Psychological characteristics				
Perceived stress	Little	4,419(72.3)	4,115(71.7)	0.663
	A lot	1,593(27.7)	1,550(28.3)	

*BMI* body mass index, *M* mean, *SE* standard error, *WC* waist circumference

*n* = 11,819

Differences in sociodemographic and lifestyle characteristics across obesity types, stratified by income level, are summarized in Tables 3 and 4. Among individuals with normal weight, the high-income group included a high proportion of women and individuals without chronic disease, but also showed less favorable dietary patterns, including more frequent breakfast skipping and higher fat intake types than other obesity types during the pandemic period (all *p* < 0.05). In those with general obesity, lifestyle patterns differed by income. In the low-income group, general obesity was more common among men and those with higher education level, more strength training, more frequent eating out, skipping breakfast, and higher fat intake than other obesity types during the pandemic period (all *p* < 0.05). In the high-income group, general obesity was common among those with higher education, more aerobic exercise, and more frequent eating out, while smoking was lower than in other obesity types during the pandemic period (all *p* < 0.05). For abdominal obesity during the pandemic period, individuals in both income groups tended to be older, have a higher prevalence of chronic disease, and have lower educational attainment. Physical activity levels were generally lower in this group, with less strength training in the low-income group and less aerobic exercise in the high-income group compared with other obesity types during the pandemic period (all *p* < 0.05). Participants with combined general and abdominal obesity showed the least favorable overall profiles. In the low-income group, combined general and abdominal obesity was characterized by higher body weight, BMI, WC, and levels of perceived stress compared with other obesity types. In the high-income group, combined general and abdominal obesity was more common among men and those with higher rates of smoking and alcohol consumption during the pandemic period (all *p* < 0.05). These findings should be interpreted with caution, as the possibility of confounding, particularly by age and sex, cannot be excluded.

**Table 3:** Differences in participant characteristics according to obesity status during the pre-pandemic and pandemic periods in low income

		Pre-pandemic					Pandemic					*P* ^*^			
		N	GO	AO	CGAO	*p*	N	GO	AO	CGAO	*p*	N	GO	AO	CGAO
		*n* (%) or M ± SE	*n* (%) or M ± SE	*n* (%) or M ± SE	*n* (%) or M ± SE		*n* (%) or M ± SE	*n* (%) or M ± SE	*n* (%) or M ± SE	*n *(%) or M ± SE					
Age (years)		51.63 ± 0.85	44.42 ± 1.63	67.36 ± 1.07	55.73 ± 0.95	< 0.001	52.66 ± 1.12	42.20 ± 2.05	67.09 ± 1.48	55.54 ± 0.99	< 0.001	0.480	0.409	0.887	0.895
Height (cm)		163.05 ± 0.37	164.14 ± 1.00	161.78 ± 0.79	173.74 ± 0.45	0.090	162.18 ± 0.42	164.09 ± 1.05	163.34 ± 0.82	164.14 ± 0.54	0.013	0.130	0.975	0.179	0.583
Body weight (kg)		50.73 ± 0.30	70.44 ± 0.87	62.64 ± 0.62	76.81 ± 0.68	< 0.001	56.79 ± 0.38	70.11 ± 0.95	63.84 ± 0.71	76.96 ± 0.70	< 0.001	0.127	0.799	0.210	0.884
BMI (kg/m^2^)		21.56 ± 0.06	26.06 ± 0.07	23.85 ± 0.08	28.51 ± 0.16	< 0.001	21.54 ± 0.09	25.97 ± 0.09	23.83 ± 0.08	28.40 ± 0.15	< 0.001	0.783	0.447	0.866	0.646
WC		77.62 ± 0.23	84.48 ± 0.36	89.98 ± 0.18	96.41 ± 0.33	< 0.001	77.42 ± 0.31	85.61 ± 0.39	90.38 ± 0.24	96.53 ± 0.36	< 0.001	0.624	0.038	0.188	0.797
Sex	Man	618(45.1)	77(60.9)	122(43.1)	350(50.3)	0.010	493(43.8)	70(64.4)	111(51.6)	360(51.9)	0.001	0.617	0.643	0.100	0.616
	Woman	866(54.9)	58(39.1)	189(56.9)	476(49.7)		721(56.2)	50(35.6)	158(48.4)	447(48.1)					
Education	≤ ES	373(20.4)	23(10.2)	151(47.8)	311(33.3)	< 0.001	288(20.7)	20(10.5)	110(43.8)	256(28.8)	< 0.001	0.825	0.411	0.847	0.567
	MS	163(10.0)	20(13.7)	45(14.0)	102(11.1)		139(11.1)	13(6.2)	36(15.2)	99(12.6)					
	HS	488(38.0)	50(45.6)	59(24.5)	219(32.9)		380(39.2)	44(46.9)	50(28.1)	213(34.0)					
	≥ College	349(31.5)	36(30.6)	28(13.6)	130(22.7)		270(29.1)	36(36.4)	21(12.9)	138(24.6)					
Marital status	Married	894(75.4)	84(87.0)	198(70.4)	520(74.8)	0.044	710(75.4)	62(74.0)	161(70.0)	477(71.7)	0.476	0.994	0.094	0.938	0.345
	Unmarried	334(24.6)	17(13.0)	103(29.6)	212(25.2)		288(24.6)	25(26.0)	95(30.0)	220(28.3)					
Region	Urban	1,134(82.1)	115(87.6)	212(72.6)	593(74.4)	< 0.001	884(80.4)	92(83.7)	186(76.9)	601(81.6)	0.498	0.745	0.584	0.593	0.233
	Rural	350(17.9)	20(12.4)	99(27.4)	233(25.6)		330(19.6)	28(16.3)	83(23.1)	206(18.4)					
Chronic disease	No	734(56.1)	61(56.3)	68(25.9)	198(30.5)	< 0.001	595(59.5)	60(59.7)	49(24.7)	187(29.1)	< 0.001	0.244	0.691	0.836	0.671
	Yes	687(43.9)	68(43.7)	235(74.1)	603(69.5)		552(40.5)	55(40.3)	210(75.3)	598(70.9)					
Smoking	No	1,185(80.1)	107(80.0)	265(85.2)	684(78.8)	0.368	996(81.3)	90(78.3)	233(85.5)	676(82.0)	0.534	0.575	0.777	0.958	0.246
	Yes	277(19.9)	23(20.0)	44(14.8)	131(21.2)		204(18.7)	29(21.7)	34(14.5)	116(18.0)					
Alcohol consumption	No	761(49.1)	62(42.0)	184(56.6)	448(51.2)	0.086	702(56.0)	58(47.4)	178(62.2)	472(55.7)	0.243	0.008	0.509	0.317	0.172
	Yes	703(50.9)	69(58.0)	125(43.4)	368(48.8)		499(44.0)	61(52.6)	89(37.8)	320(44.3)					
Walking	Never	253(16.2)	19(12.3)	69(21.0)	190(24.2)	0.004	215(17.7)	15(12.6)	55(20.6)	151(20.0)	0.680	0.660	0.962	0.784	0.419
	1–2 d/wk	204(14.5)	17(13.5)	40(14.9)	101(14.4)		161(16.1)	15(10.7)	35(14.3)	103(14.1)					
	3–4 d/wk	287(20.5)	23(18.9)	42(14.5)	166(21.4)		213(20.2)	19(19.6)	36(18.6)	133(20.6)					
	5–7 d/wk	629(48.8)	70(55.3)	133(49.6)	303(40.1)		488(46.0)	63(57.0)	92(46.5)	318(45.4)					
Strength training	No	1,059(74.6)	90(68.1)	236(82.0)	627(82.7)	0.001	815(73.5)	69(54.7)	183(84.0)	570(76.8)	< 0.001	0.661	0.087	0.673	0.032
	Yes	316(25.4)	39(31.9)	49(18.0)	135(17.3)		261(26.5)	44(45.3)	35(16.0)	136(23.2)					
Aerobic exercise	No	826(57.1)	59(50.6)	186(65.4)	493(62.2)	0.038	651(59.2)	54(50.1)	150(63.6)	457(60.1)	0.309	0.490	0.947	0.762	0.536
	Yes	544(42.9)	69(49.4)	99(34.6)	267(37.8)		424(40.8)	58(49.9)	68(36.4)	247(39.9)					
Sedentary behavior	≤ 8 h	603(41.8)	60(42.0)	115(45.3)	289(28.2)	0.667	465(43.6)	45(34.2)	81(34.5)	296(41.8)	0.326	0.896	0.322	0.055	0.622
	> 8 h	759(58.2)	67(58.0)	158(54.7)	462(32.7)		602(56.4)	67(65.8)	131(65.5)	398(58.2)					
Eating out	≤3/mo	492(32.1)	33(20.6)	139(43.8)	288(46.6)	0.001	442(40.2)	32(27.3)	130(54.5)	315(42.9)	0.020	0.018	0.254	0.194	0.005
	1-6/wk	607(49.1)	58(50.3)	124(46.4)	335(20.7)		410(45.8)	49(54.8)	70(37.6)	247(44.2)					
	Every day	184(18.8)	27(29.1)	19(9.8)	107(32.7)		99(14.0)	13(17.9)	13(7.9)	64(12.9)					
Breakfast skipping	≤ 2 d/wk	889(61.7)	65(48.1)	242(83.3)	533(73.0)	< 0.001	649(60.6)	55(50.3)	184(81.2)	449(64.1)	< 0.001	0.563	0.153	0.822	0.283
	3–6 d/wk	221(21.5)	36(35.3)	25(10.5)	117(16.0)		173(24.0)	21(21.9)	16(10.6)	89(17.6)					
	Every day	173(16.8)	17(16.6)	15(6.2)	80(11.0)		129(15.3)	18(27.8)	13(8.1)	88(18.3)					
Energy intake %		87.47 ± 1.35	86.21 ± 4.08	86.54 ± 2.41	84.85 ± 1.71	0.706	84.70 ± 1.31	84.04 ± 3.33	87.05 ± 3.62	87.67 ± 2.21	0.624	0.143	0.664	0.907	0.321
Fat intake %		21.21 ± 0.38	22.38 ± 0.94	17.25 ± 0.55	19.35 ± 0.47	< 0.001	21.52 ± 0.51	25.76 ± 1.42	18.95 ± 0.79	20.82 ± 0.61	< 0.001	0.645	0.045	0.090	0.060
Carbohydrate intake %		63.67 ± 0.44	61.23 ± 1.15	67.89 ± 0.75	65.75 ± 0.61	< 0.001	63.63 ± 0.63	58.45 ± 1.56	66.22 ± 0.94	64.40 ± 0.78	< 0.001	0.965	0.151	0.181	0.170
Perceived stress	Little	1,071(74.1)	93(71.5)	238(73.2)	611(75.0)	0.781	887(72.5)	84(70.7)	221(85.4)	573(69.6)	0.010	0.528	0.910	0.006	0.486
	A lot	389(25.9)	38(28.5)	71(26.8)	204(25.0)		311(27.5)	35(29.3)	45(14.6)	218(30.4)					

N normal, GO general obesity, AO abdominal obesity, CGAO combined general and abdominal obesity, ES elementary school, MS middle school, HS high school, d day, wk week

Low-income group (*n* = 5,166)

* Within-group comparison between 2019 and 2020

**Table 4:** Differences in participant characteristics according to obesity status during the pre-pandemic and pandemic periods in high income

Variables		Pre-pandemic					Pandemic					*P* ^*^			
		N	GO	AO	CGAO	*p*	N	GO	AO	CGAO	*p*	N	GO	AO	CGAO
		*n* (%) or M ± SE	*n* (%) or M ± SE	*n* (%) or M ± SE	*n* (%) or M ± SE		*n (%) or M ± SE*	*n (%) or M ± SE*	*n (%) or M ± SE*	*n* (%) or M ± SE					
Age (years)		42.50 ± 0.44	40.71 ± 0.90	56.83 ± 1.12	47.97 ± 0.64	< 0.001	42.89 ± 0.50	42.84 ± 1.06	56.79 ± 1.26	47.14 ± 0.64	< 0.001	0.569	0.128	0.983	0.356
Height (cm)		165.62 ± 0.23	166.76 ± 0.67	166.45 ± 0.89	168.41 ± 0.44	< 0.001	165.29 ± 0.29	165.68 ± 0.64	167.59 ± 0.84	168.70 ± 0.42	< 0.001	0.359	0.242	0.378	0.638
Body weight (kg)		59.51 ± 0.25	72.56 ± 0.61	66.76 ± 0.73	80.46 ± 0.56	< 0.001	59.73 ± 0.29	71.86 ± 0.64	67.95 ± 0.74	81.15 ± 0.57	< 0.001	0.572	0.430	0.273	0.383
BMI (kg/m^2^)		21.61 ± 0.06	26.02 ± 0.07	24.01 ± 0.07	28.27 ± 0.12	< 0.001	21.76 ± 0.06	26.11 ± 0.10	24.11 ± 0.07	28.42 ± 0.15	< 0.001	0.082	0.462	0.314	0.447
WC		76.72 ± 0.20	85.23 ± 0.28	90.00 ± 0.33	96.06 ± 0.32	< 0.001	76.88 ± 0.20	85.32 ± 0.25	90.25 ± 0.28	96.06 ± 0.30	< 0.001	0.566	0.813	0.575	0.998
Sex	Man	795(44.7)	143(64.8)	87(48.1)	517(65.7)	< 0.001	717(41.4)	140(61.3)	95(58.1)	595(66.6)	< 0.001	0.074	0.514	0.145	0.738
	Woman	1,228(55.3)	97(35.2)	105(51.9)	338(34.3)		1,151(58.6)	116(38.7)	88(41.9)	385(33.4)					
Education	≤ ES	82(2.8)	9(1.8)	31(16.6)	73(8.9)	< 0.001	76(3.2)	9(3.7)	23(11.0)	78(4.8)	< 0.001	0.446	0.485	0.070	0.375
	MS	108(4.3)	13(5.4)	19(8.8)	79(9.6)		110(4.3)	10(3.3)	35(18.4)	73(6.0)					
	HS	676(35.5)	79(35.3)	63(32.3)	288(35.0)		667(38.5)	94(38.8)	56(38.2)	341(37.4)					
	≥ College	1,079(57.3)	132(57.5)	69(42.3)	383(46.5)		919(54.0)	124(54.3)	55(32.4)	445(51.7)					
Marital status	Married	1,469(94.7)	171(95.0)	153(85.3)	671(93.4)	0.001	1,231(91.8)	177(94.9)	159(88.3)	717(92.2)	0.245	0.012	0.961	0.466	0.472
	Unmarried	106(5.3)	7(5.0)	30(14.7)	63(6.6)		121(8.2)	13(5.1)	20(11.7)	73(7.8)					
Region	Urban	1,749(89.9)	192(87.3)	168(86.6)	696(85.3)	0.016	1,597(91.2)	220(91.1)	149(84.6)	808(89.0)	0.080	0.671	0.337	0.778	0.357
	Rural	274(10.1)	48(12.7)	24(13.4)	159(14.7)		271(8.8)	36(8.9)	34(15.4)	172(11.0)					
Chronic disease	No	1,371(69.7)	152(69.5)	63(39.5)	338(44.5)	< 0.001	1,240(72.6)	147(63.4)	58(38.1)	382(43.8)	< 0.001	0.583	0.243	0.830	0.808
	Yes	595(30.3)	84(30.5)	121(60.5)	493(55.5)		560(27.4)	104(36.6)	116(61.9)	574(56.2)					
Smoking	No	1,695(82.8)	195(79.0)	152(76.0)	648(75.1)	0.002	1,572(84.0)	220(85.3)	152(79.5)	779(77.3)	0.010	0.555	0.130	0.538	0.428
	Yes	308(17.2)	43(21.0)	34(24.0)	194(24.9)		292(16.0)	35(14.7)	31(20.5)	192(22.7)					
Alcohol consumption	No	790(37.4)	97(40.8)	83(45.2)	328(36.6)	0.308	789(41.0)	99(34.8)	92(46.8)	371(34.7)	0.029	0.108	0.253	0.807	0.531
	Yes	1,213(62.6)	141(59.2)	103(54.8)	516(63.4)		1,075(59.0)	156(65.2)	91(53.2)	600(65.3)					
Walking	Never	271(12.8)	32(14.4)	39(16.9)	137(16.0)	0.269	259(14.0)	33(10.8)	30(16.9)	169(17.0)	0.739	0.005	0.814	0.917	0.056
	1–2 d/wk	319(14.2)	41(18.3)	25(17.7)	119(12.6)		326(18.9)	44(20.2)	27(17.8)	155(18.4)					
	3–4 d/wk	395(19.1)	52(19.8)	40(19.5)	181(21.7)		364(20.7)	51(20.3)	44(23.0)	193(21.0)					
	5–7 d/wk	960(53.8)	108(47.6)	78(45.9)	386(49.7)		823(46.4)	109(48.7)	68(42.2)	422(43.6)					
Strength training	No	1,368(67.6)	153(62.6)	146(80.9)	605(73.1)	0.002	1,242(68.9)	156(64.8)	124(69.7)	686(71.1)	0.502	0.516	0.672	0.058	0.477
	Yes	577(32.4)	80(37.4)	36(19.1)	218(26.9)		530(31.1)	81(35.2)	45(30.3)	253(28.9)					
Aerobic exercise	No	1,009(49.5)	112(45.2)	107(60.7)	456(54.8)	0.022	969(52.6)	108(42.2)	110(67.6)	509(53.6)	0.002	0.159	0.601	0.317	0.726
	Yes	937(50.5)	121(54.8)	75(39.3)	367(45.2)		803(47.4)	129(57.8)	59(32.4)	430(46.4)					
Sedentary behavior	≤ 8 h	764(36.2)	87(31.7)	67(38.8)	322(35.8)	0.644	708(37.8)	88(30.3)	75(40.9)	361(35.0)	0.194	0.542	0.823	0.757	0.834
	> 8 h	1,176(63.8)	145(68.3)	113(61.2)	495(64.2)		1,060(62.2)	149(69.7)	94(59.1)	576(65.0)					
eating out	≤3/mo	259(12.0)	26(11.2)	51(29.6)	122(13.9)	< 0.001	288(16.9)	34(12.7)	45(27.2)	140(15.6)	0.030	< 0.001	0.387	0.812	0.133
	1-6/wk	992(55.1)	94(45.0)	80(45.0)	361(50.2)		798(55.6)	96(51.4)	71(49.2)	400(54.3)					
	Every day	507(32.9)	89(43.7)	35(25.4)	229(35.9)		361(27.4)	62(35.9)	24(23.6)	202(30.1)					
Breakfast skipping	≤ 2 d/wk	992(52.3)	101(44.9)	125(71.1)	408(52.7)	0.004	744(46.1)	91(44.9)	109(73.7)	427(51.3)	< 0.001	0.014	0.392	0.850	0.170
	3 − 6 d/wk	431(26.3)	64(33.5)	27(17.6)	176(27.3)		348(26.3)	46(27.8)	17(14.6)	160(23.8)					
	Every day	335(21.4)	44(21.6)	14(11.3)	128(20.0)		355(27.6)	55(27.3)	14(11.8)	155(24.8)					
Energy intake %		91.27 ± 1.15	95.20 ± 2.68	97.14 ± 3.64	95.10 ± 1.98	0.127	90.89 ± 1.20	85.89 ± 3.18	89.83 ± 3.46	95.17 ± 1.85	0.082	0.818	0.023	0.119	0.979
Fat intake %		24.16 ± 0.27	25.85 ± 0.93	20.25 ± 0.74	23.66 ± 0.40	< 0.001	25.71 ± 0.33	24.02 ± 0.81	23.18 ± 0.95	24.23 ± 0.43	0.001	< 0.001	0.138	0.017	0.342
Carbohydrate intake %		59.90 ± 0.35	56.95 ± 1.16	64.36 ± 0.92	60.40 ± 0.51	< 0.001	58.41 ± 0.37	59.67 ± 0.87	60.67 ± 1.03	59.53 ± 0.48	0.056	0.004	0.061	0.009	0.228
Perceived stress	Little	1,489(73.5)	174(72.3)	142(72.2)	582(67.2)	0.056	1,321(70.9)	195(80.3)	138(74.2)	683(69.2)	0.052	0.167	0.100	0.749	0.523
	A lot	514(26.5)	64(27.7)	44(27.8)	261(32.8)		543(29.1)	60(19.7)	45(25.8)	288(30.8)					

N normal, GO general obesity, AO abdominal obesity, CGAO combined general and abdominal obesity, ES elementary school, MS middle school, HS high school, d day, wk week

High-income group (*n* = 6,653)

* Within-group comparison between 2019 and 2020

Tables 3 and 4 also showed differences in lifestyle behaviors in the pre-pandemic and pandemic period according to income level and obesity phenotype. In the low-income group, several lifestyle and dietary variables differed according to obesity phenotype when comparing the pre-pandemic and pandemic periods. Individuals with normal weight tended to report lower alcohol consumption, whereas strength training was more common among those with combined general and abdominal obesity during the pandemic. High perceived stress was less frequently reported in the abdominal obesity group. Eating out was less common in the normal-weight and combined general and abdominal obesity groups, while fat intake was relatively higher in the general obesity group during the pandemic period than during the pre-pandemic period.

In the high-income group, energy intake was lower in the general obesity group, and eating out was less frequent in the normal-weight group, and walking was less common among individuals with normal weight, who also showed a higher frequency of skipping breakfast during the pandemic period than during the pre-pandemic period. Fat intake was higher in the normal-weight and abdominal obesity groups, whereas carbohydrate intake was lower in these groups during the pandemic period than during the pre-pandemic period (all *p* < 0.05).

To examine variables associated with combined general and abdominal obesity, logistic regression analysis were conducted separately by income level (Table 5). All models were adjusted for age and sex. In the low-income group, combined general and abdominal obesity was more common among older adults, men, individuals with lower education levels, and those with chronic diseases in both the pre-pandemic and pandemic periods. In the pre-pandemic period, excessive carbohydrate intake, walking, and strength training were associated with combined general and abdominal obesity; however, the associations were not significant in the pandemic-period., In the high-income group, combined general and abdominal obesity was more likely among middle-aged and older men and individuals with chronic diseases (OR: 3.04; 95% CI: 2.38–3.88; *p* < 0.001). Moreover, lack of strength training (OR: 1.34; 95% CI: 1.03–1.74; *p* = 0.029), higher total energy intake (OR: 1.58; 95% CI: 1.16–2.16; *p* = 0.004), and higher perceived stress (OR: 1.27; 95% CI: 1.02–1.59; *p* = 0.033) were positively associated with combined general and abdominal obesity in this group in the pandemic period.

**Table 5:** Logistic regression analysis for the prevalence of combined general and abdominal obesity according to lifestyle by income group

Variable	Characteristics	Pre-pandemic			Pandemic		
		AOR	95% CI	*P*	AOR	95% CI	*P*
Low-income group							
Age(years)^1)^	Older adults (ref. young adults)	1.68	(1.28–2.21)	< 0.001	1.49	(1.05–2.12)	0.026
	Middle-aged adults (ref. young adults)	1.29	(0.98–1.69)	0.066	1.28	(0.91–1.81)	0.153
Sex^2)^	Man (ref. woman)	1.26	(1.01–1.58)	0.044	1.42	(1.10–1.83)	0.007
Education	≤ Middle school (ref. ≥ high school)	1.67	(1.26–2.22)	< 0.001	1.53	(1.06–2.21)	0.024
Marital status	Unmarried (ref. married)	0.93	(0.72–1.20)	0.576	1.23	(0.89–1.70)	0.199
Region	Rural (ref. urban)	1.46	(1.17–1.82)	0.001	0.85	(0.66–1.10)	0.219
Chronic disease	Yes (ref. no)	3.00	(2.24–4.02)	< 0.001	4.48	(3.18–6.31)	< 0.001
Smoking	Yes (ref. no)	1.09	(0.79–1.51)	0.582	0.86	(0.65–1.15)	0.314
Alcohol consumption	Yes (ref. no)	0.95	(0.76–1.18)	0.630	0.98	(0.77–1.26)	0.900
Walking	No (ref. yes)	1.55	(1.22–1.96)	< 0.001	1.09	(0.78–1.52)	0.609
Strength training	No (ref. yes)	1.70	(1.26–2.28)	< 0.001	1.28	(0.97–1.71)	0.085
Aerobic exercise	No (ref. yes)	1.14	(0.91–1.42)	0.255	0.99	(0.77–1.28)	0.958
Sedentary behavior	> 8 h (ref. ≤ 8 h)	0.91	(0.72–1.15)	0.414	0.98	(0.76–1.28)	0.906
Eating out	Every day (ref. ≤ 3 times or less/month)	1.49	(1.03–2.16)	0.036	0.99	(0.62–1.59)	0.977
	1–6 times / week (ref. ≤ 3 times or less/month)	1.13	(0.90–1.41)	0.290	1.01	(0.73–1.40)	0.941
Breakfast skipping	Every day (ref. < 2 times / week)	1.19	(0.78–1.82)	0.412	1.35	(0.94–1.95)	0.108
	3–6 days per week (ref. < 2 times / week)	1.40	(1.00-1.98)	0.053	0.83	(0.56–1.23)	0.355
Energy intake	Deficient (ref. adequate)	1.09	(0.85–1.41)	0.490	1.02	(0.75–1.38)	0.895
	Excessive (ref. adequate)	0.95	(0.61–1.49)	0.820	1.25	(0.75–2.07)	0.393
Fat intake	Deficient (ref. adequate)	1.24	(0.93–1.64)	0.138	0.95	(0.72–1.25)	0.708
	Excessive (ref. adequate)	0.96	(0.7–1.31)	0.776	0.94	(0.65–1.36)	0.744
Carbohydrate intake	Deficient (ref. adequate)	1.14	(0.81–1.61)	0.448	0.88	(0.62–1.26)	0.490
	Excessive (ref. adequate)	1.34	(1.03–1.74)	0.030	1.00	(0.74–1.36)	0.982
Perceived stress	A lot (ref. little)	1.25	(0.97–1.62)	0.084	1.28	(0.96–1.71)	0.094
High-income group							
Age (years)^1)^	Elderly (ref. young adults)	2.96	(2.10–4.18)	< 0.001	2.28	(1.58–3.28)	< 0.001
	Middle-aged (ref. young adults)	1.94	(1.57–2.41)	< 0.001	1.64	(1.31–2.06)	< 0.001
Gender^2)^	Man (ref. woman)	2.48	(2.00-3.06)	< 0.001	2.89	(2.33–3.59)	< 0.001
Education	≤ Middle school (ref. ≥ high school)	1.52	(1.09–2.14)	0.015	1.19	(0.85–1.67)	0.306
Marriage status	Unmarried (ref. married)	1.27	(0.83–1.95)	0.270	0.93	(0.61–1.40)	0.710
Region of residence	Rural (ref. urban)	1.36	(1.04–1.80)	0.028	1.20	(0.95–1.53)	0.127
Chronic disease	Yes (ref. no)	2.97	(2.38–3.71)	< 0.001	3.04	(2.38–3.88)	< 0.001
Smoking	Yes (ref. no)	1.21	(0.92–1.60)	0.181	1.01	(0.71–1.38)	0.925
Alcohol consumption	Yes (ref. no)	0.92	(0.72–1.17)	0.493	1.14	(0.87–1.50)	0.343
Walking	No (ref. yes)	1.19	(0.89–1.59)	0.247	1.11	(0.82–1.50)	0.505
Strength training	No (ref. yes)	1.48	(1.16–1.89)	0.002	1.34	(1.03–1.74)	0.029
Aerobic exercise	No (ref. yes)	1.23	(1.00-1.52)	0.045	1.06	(0.86–1.31)	0.571
Sedentary behavior	> 8 h (ref. ≤ 8 h)	0.93	(0.73–1.18)	0.525	0.84	(0.67–1.06)	0.136
Eating out	Every day (ref. ≤ 3 times / month)	1.04	(0.75–1.45)	0.802	1.07	(0.76–1.50)	0.699
	1–6 times / week (ref. ≤ 3 times / month)	0.94	(0.71–1.25)	0.680	1.18	(0.86–1.64)	0.309
Breakfast skipping	Every day (ref. ≤ 2 days / week)	1.39	(1.03–1.88)	0.034	1.08	(0.82–1.42)	0.563
	3–6 days / week (ref. ≤ 2days / week)	1.50	(1.14–1.95)	0.003	1.07	(0.81–1.41)	0.652
Energy intake	Deficient (ref. adequate)	1.04	(0.81–1.34)	0.759	1.29	(0.97–1.71)	0.083
	Excessive (ref. adequate)	1.23	(0.87–1.75)	0.242	1.58	(1.16–2.16)	0.004
Fat intake	Deficient (ref. adequate)	0.93	(0.71–1.23)	0.627	0.85	(0.64–1.14)	0.277
	Excessive (ref. adequate)	1.20	(0.93–1.54)	0.155	0.79	(0.59–1.05)	0.103
Carbohydrate intake	Deficient (ref. adequate)	1.18	(0.92–1.51)	0.203	0.99	(0.73–1.35)	0.945
	Excessive (ref. adequate)	0.90	(0.71–1.15)	0.410	1.08	(0.85–1.35)	0.535
Perceived stress	A lot (ref. little)	1.74	(1.40–2.16)	< 0.001	1.27	(1.02–1.59)	0.033

AORs were estimated using complex-sample logistic regression models fitted separately for each lifestyle factor. Each model was adjusted for age and sex

1) age was adjusted for sex; 2) sex was adjusted for age

*AOR* adjusted odds ratio,* CI* confidence interval. Adjusted for age and gender

AORs (95% CI)

## Discussion

In our study, we showed that lifestyle patterns in 2020 differed from those in 2019, with divergent across income and obesity groups. During the pandemic period (2020), certain low-income subgroups exhibited higher rates of strength training and lower alcohol consumption compared with the pre-pandemic period (2019). In contrast, the high-income group during the pandemic period showed a higher prevalence of breakfast skipping and lower walking compared with the same group during the pre-pandemic period. During the pandemic period the variables associated with combined general and abdominal obesity also varied by income. In the low-income group, older age, male sex, lower education, and chronic diseases were key variables. In the high-income group, middle-to-older age, male sex, chronic diseases, lack of strength training, perceived stress, and excessive energy intake were significantly associated with combined general and abdominal obesity.

Notably, the prevalence of smoking (high-income) and chronic diseases was lower among those with general obesity compared with other obesity groups. This is consistent with cohort data showing that cardiovascular risk is higher in abdominal and combined general and abdominal obesity phenotypes than in general obesity alone, as demonstrated in Chinese [11] and Korean adult populations [12] .

Our focus on combined general and abdominal obesity is supported by the growing consensus that single indicators such as BMI or WC are insufficient to evaluate obesity and its associated metabolic outcomes [31, 32]. Recent study demonstrates that certain anthropometric markers are more sensitive in identifying cardiometabolic risk within vulnerable population in prison than BMI alone, suggested that the importance of using diverse indices to manage chronic diseases [31]. Obesity must be viewed as a qualitative metabolic state rather than just a quantitative weight issue, therefore, utilizing multiple anthropometric indicators allows for a low-cost, non-invasive, highly effective method of identifying “at-risk” individuals who might otherwise be classified as “healthy” by BMI standards [31, 32]. Besides, an integrated approach evaluating modifiable lifestyle behaviors—including physical activity, dietary patterns, and psychosocial variables—alongside obesity phenotypes is essential, because it is a gateway to understanding the obesity type [33, 34].

First, we found that smoking and alcohol consumption were higher in combined general and abdominal obesity than in other obesity types at high-income levels, while participants with abdominal obesity had lower physical activities, such as strength training (low income) or aerobic exercise (high income), than other obesity types during the pandemic. In a previous study, the number of days of walking and strength training was higher in the non-abdominal obesity group than in the abdominal obesity group after the COVID-19 pandemic [35], consistent with the results of strength training in the low-income group in this study (normal: 26.5%; abdominal obesity: 16.0%) during the pandemic. BMI is the most commonly used adiposity indicator, but body fat distribution, particularly central accumulation measured by WC, is a stronger predictor of cardiometabolic risk [9]. In our study, health-related indicators in individuals with combined general and abdominal obesity exhibited a worse profile than those with normal weight or other obesity patterns during the pandemic period. This finding is consistent with prospective cohort data showing that combined general and abdominal obesity confers the highest risk of incident cardiovascular disease compared with other BMI–WC phenotypes [11].

Second, when comparing lifestyle during pre-pandemic and pandemic period, our results revealed that monthly drinking in the normal-weight group was lower in the low-income during the pandemic than pre-pandemic period. The pandemic environment may have reduced alcohol consumption by restricting social interactions [35] or increased it due to stress [36]. In addition, while physical activity levels generally decreased globally [37], our study showed that walking was lower during the pandemic than pre-pandemic period in the high-income normal-weight group, but strength training was higher during the pandemic than pre-pandemic period in the low-income combined general and abdominal obesity group which may be because it could be performed at home despite social distancing and gym closures [21, 38, 39].

Third, in the high-income group, lack of strength training, perceived stress, and excessive energy intake were positively associated with combined general and abdominal obesity during the pandemic period, consistent with findings that lack of physical activity relates to obesity prevalence [35]. A national survey in South Korea found that not engaging in physical activity was associated with general and abdominal obesity among middle-aged and older Korean women, suggesting that lack of exercise may reduce overall energy expenditure, which could result in a positive energy balance [25]. Additionally, several studies reported that mental health and energy intake are strongly linked to obesity which is consistent with our findings [40, 41].

Our study also identified differences in lifestyles associated with combined general and abdominal obesity according to income level. In the low-income group, excessive carbohydrate intake was associated with combined general and abdominal obesity during pre-pandemic period, consistent with poor nutrition from processed grains [42]. In the high-income group, breakfast skipping and excessive energy intake were notable. While breakfast skipping increased during the pandemic [43], frequent overeating due to changed eating patterns also influenced obesity [23], suggesting a need for management programs that reflect these specific variables in high-income groups.

During the pandemic, restrictions such as lockdowns were associated with reduced physical activity, increased sedentary time, and changes in eating behaviors [20–23], which may have varied by socioeconomic status. These income-specific disparities suggest that unequal environmental resources during the pandemic period were related to divergent pathways to obesity. Günal et al. [33] has noted that individuals of lower socioeconomic status are in a global surge in poor health profiles due to restricted access to fresh food and safe exercise environments. In the high-income group, the profile was characterized by a clustering of behavioral distortions, such as excessive energy intake, lack of strength training, perceived stress, which results suggest that diet and activity operate through different mechanisms based on socioeconomic status. These behavioral patterns may be linked to differing levels of obesity awareness and health literacy. Selcuk et al. [34] demonstrated that increased awareness and motivation can reduce food cravings and improve metabolic parameters; moreover, high-income groups may benefit from personalized digital self-monitoring to enhance health literacy. Conversely, the disparities observed in our study align with global health vulnerability patterns [33], where socioeconomic constraints create distinct risk profiles.

Consequently, public health strategies should move toward income-sensitive interventions. For the low-income group, priorities should include enhancing healthy food accessibility through nutritional education and subsidies [44] and providing low-cost community exercise programs [45]. For the high-income group, strategies may need to focus on leveraging digital health technologies for personalized self-monitoring, alongside environmental strategies of health-friendly choices in dining-out settings [46, 47]. National obesity policies should be designed as tailored strategies that account for the socioeconomic distribution of health determinants and the practical feasibility within each income stratus.

The results of this study are relevant because we used nationally representative KNHANES data and focused on modifiable lifestyle variables, providing valuable insights into how these variables related to combined general and abdominal obesity, particularly during the pandemic. In addition, our findings highlight income-stratified differences in combined general and abdominal obesity and offer new perspectives on population-level trends.

This study has several limitations. First, this was a cross-sectional study that did not exclude participants with chronic diseases, which precludes inferences of causation; the possibility of reverse causation remains. Second, despite adjusting for age and sex, residual confounding may persist due to unmeasured environmental variables in the KNHANES, which precluded accounting for work-from-home status, or facility closures, which may have influenced both lifestyle behaviors and obesity outcomes, and thus the findings should be interpreted with caution. Third, we could not isolate the effects of specific, time-bound policy measures because the KNHANES data are collected throughout the year and pandemic restrictions varied across 2020.

## Conclusions

These results suggest that modifiable behavioral variables such as physical activity, dietary variables, and stress were associated with combined general and abdominal obesity in high-income level during the pandemic period. These findings underscore the importance of addressing modifiable behavioral variables as core strategies for obesity prevention. Therefore, sustainable, income-sensitive strategies to promote healthy lifestyles is needed in the post-pandemic era. Tailored interventions that account for both socioeconomic status and specific obesity phenotypes, particularly combined general and abdominal obesity, are crucial for mitigating long-term health impacts and reducing the prevalence of high-risk obesity complications.

## Data Availability

The authors do not have permission to share data.

## Abbreviations

BMI: Body mass index
CI: Confidence interval
KNHANES: Korea National Health and Nutrition Examination Survey
OR: Odds ratio
SE: Standard error
WC: Waist circumference
